# Oncolytic viruses: Revolutionising the fight against tumours

**DOI:** 10.1002/ctm2.70503

**Published:** 2025-10-15

**Authors:** Zeda Zhao, Yinan Shen, Tingbo Liang

**Affiliations:** ^1^ Oncolytic Virus Innovation Research Group Zhejiang Provincial Key Laboratory of Pancreatic Disease Hangzhou China; ^2^ Department of Hepatobiliary and Pancreatic Surgery The First Affiliated Hospital Zhejiang University School of Medicine Hangzhou China

1

Oncolytic virotherapy has completed a century‐long translational odyssey from conceptual hypothesis to therapeutic. As early as 1904, scientists observed cases where influenza infection caused a rapid decline in patients' leukocyte counts.^1^ After prolonged development, it wasn't until the 1990s that genetically engineered oncolytic viruses with attenuated backbones were successfully developed.^2^ In the field of oncolytic virotherapy, product iterations demonstrate distinct generational evolution characteristics: The first‐generation products represented by Oncorine® primarily relied on the virus's intrinsic lytic effects. Due to the lack of exogenous genetic modifications, these agents often failed to elicit robust systemic anti‐tumour immune responses. Early‐phase clinical trials of granulocyte‐macrophage colony‐stimulating factor (GM‐CSF)‐expressing oncolytic virus JX‐594 (Pexa‐Vec) established its safety profile, yet subsequent Phase II studies revealed limited therapeutic durability.^3^ The observed clinical paradox may be attributed to the pleiotropic nature of GM‐CSF, a cytokine capable of exerting bidirectional immunoregulatory effects that simultaneously stimulate and inhibit anti‐tumour immunit.^4^, ^5^, ^6^, ^7^ Concurrently, the past decade has seen a surge in research efforts across academic and industrial sectors dedicated to advancing oncolytic virotherapy, accompanied by innovative engineering of next‐generation oncolytic viral platforms. While oncolytic virotherapy has achieved clinical success in select solid malignancies (e.g., HCC,^8^ melanoma,^9^ glioma,^10^) its limited activity in treatment‐resistant populations‐particularly those with immunologically ‘cold’ tumours such as pancreatic cancer‐highlights three critical research imperatives: (i) deciphering the precise mechanisms of viral‐mediated oncolysis, (ii) systematically characterising resistance pathways, and (iii) developing rationally designed combination regimens.

As a form of tumour immunotherapy, oncolytic viruses not only directly lyse and kill tumour cells but also fundamentally aim to stimulate and mobilise the anti‐tumour immune response, thereby effectively combating and suppressing tumours. A promising strategy to enhance the therapeutic potential of oncolytic viruses is to engineer them to carry immune‐stimulating factors. The oncolytic virus product we developed, VG161, is a next‐generation oncolytic virus that carries immune‐stimulating factors (IL‐12, IL‐15/IL‐15Rα) and a PD‐L1 blocker.

Multi‐therapeutic payloads engineered on anti‐tumour immunologic principles. In addition to the conventional anti‐tumour mechanisms of oncolytic viruses (Figure 1, left), ​VG161​ carries multiple immune‐stimulating factors to enhance anti‐tumour immune activation and improve efficacy, thereby ​re‐establishing the anti‐tumour immune cycle in drug‐resistant liver cancer (Figure 1, right): (i) IL‐12 strengthens the ability of ​CD8⁺ T cells​ to recognise and kill tumour cells while partially activating ​NK cells; (ii) IL‐15​ significantly promotes the proliferation of ​NK cells​ and ​memory CD8⁺ T cells, improving innate immune activity and immune persistence; and (iii) the ​PD‐L1 antagonistic peptide​ blocks the immune checkpoint pathway, relieves immunosuppression, and reverses ​T‐cell exhaustion. These three components target different nodes of the immune cycle: IL‐12​ initiates immunity, IL‐15​ sustains activity and PD‐L1 blockade​ removes suppression. By acting on the ​initiation, expansion, and effector phases​ of immunity, they form a ​closed‐loop synergy, leading to ​enhanced therapeutic effects.

**FIGURE 1:**
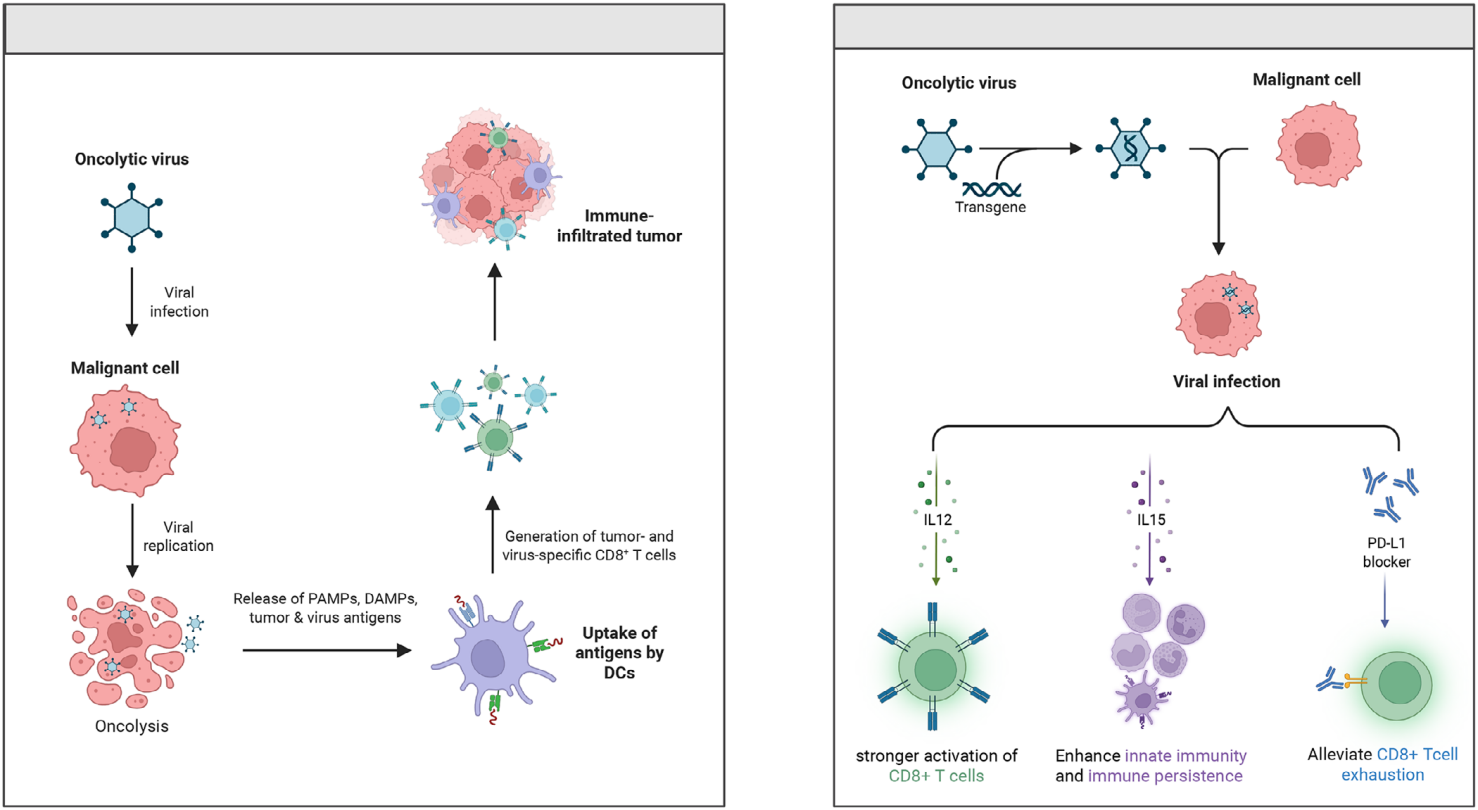
Comprehensive schematic of VG161 anti‐tumour mechanisms. Figure created using biorender.com.

The clinical application potential and prospective design of the oncolytic virus in our study. (1) ​Safety: No dose‐limiting toxicity was observed even at a total dose of up to 5.0 × 10⁸ PFU. In mice models, VG161 performed rapid replications in tumour tissues and high efficiency in killing malignancies, meanwhile no active virus was detected in the liver, spleen, heart, lungs, or blood. This fully confirms that VG161 can specifically infect and replicate within tumour cells without attacking normal tissues and cells. (2) ​New Hope for Advanced Hepatocellular Carcinoma (HCC) Patients after Second‐Line Treatment Failure: VG161 has shown excellent clinical efficacy in HCC patients. Among 37 evaluable patients, the objective response rate (ORR) was 18.92% (7/37), and the disease control rate (DCR) was 64.86% (24/37). The median progression‐free survival (PFS) was 2.9 months, and the median overall survival (OS) was 12.4 months. Furthermore, patients who had previously undergone various treatments (including immunotherapy and kinase inhibitors) appeared to respond better to VG161, suggesting that in future clinical applications, VG161 may synergise with existing standard treatment regimens. (3) The observed abscopal responses: prominent shrinkage in non‐injected tumour lesions. As a matter of fact, multicolour immunofluorescence, single‐cell sequencing, and mouse animal experiments collaboratively illustrated that VG161 substantially enhance the infiltration of anti‐tumour T cells in the tumour microenvironment and boost their anti‐tumour immune activity from various perspectives, ultimately leading to remarkable improvement in reversing the immunosuppressive tumour microenvironment. This, to some extent, explains why patients with prior immunotherapy experience responded better to VG161.

Combinatorial OV strategies are becoming central to advancing tumour immunotherapy (Figure 2). (1) Screening and optimisation of tumour‐specific oncolytic viral backbone: The backbone of an oncolytic virus determines its replication characteristics, tissue tropism, safety, and immunogenicity. Detecting and analysing the receptor expression of different oncolytic virus backbones in tumours enables the selection of the optimal backbone virus, thereby maximising the infection, replication, and oncolytic efficacy of the oncolytic virus. (2) Cytokine‐Armed Oncolytic Viruses: Addressing tumour heterogeneity through in‐depth analysis of key regulators in the tumour immune microenvironment, and utilising oncolytic viral vectors to express or block relevant factors, thereby maximising systemic anti‐tumour immunity. (3) Combination therapy with other immunotherapies. The application of cell therapy in solid tumours remains challenging, largely owing to insufficient tumour‐specific antigen presentation that impairs T‐cell recognition and cytotoxicity. To overcome this limitation, genetically modified oncolytic viruses encoding tumour‐specific antigens can serve a dual function: while lysing tumour cells, these viruses simultaneously provide targetable antigens for subsequent T‐cell therapy.

**FIGURE 2:**
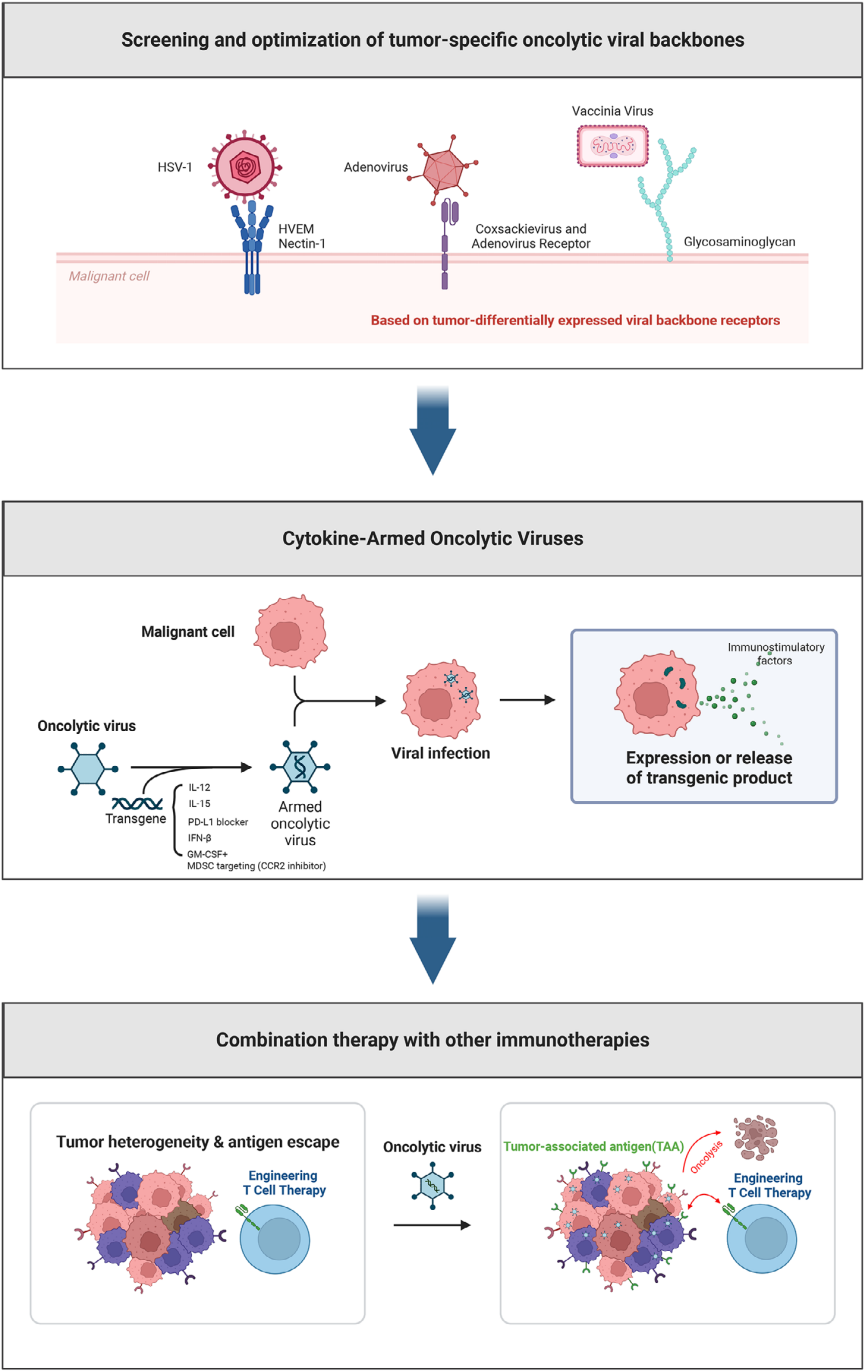
Engineering strategies for oncolytic viruses and their combination with other immunotherapies. Figure created using biorender.com.

## CONFLICT OF INTEREST STATEMENT

The authors declare that they have no competing interests.
